# Mapping between EQ-5D-5L and EQ-5D-3L in lumbar spine surgery patients: Machine learning compared to crosswalk algorithms

**DOI:** 10.1016/j.bas.2026.107459

**Published:** 2026-09-28

**Authors:** Natalie Hong Siu Chang, Casper Friis Pedersen, Mikkel Østerheden Andersen, Leah Yacat Carreon

**Affiliations:** a Medical Spinal Research Unit, Spine Centre of Southern Denmark, Lillebaelt Hospital - University Hospital of Southern Denmark, Sygehusvej 24, Kolding, 6000, Denmark; b Institute of Regional Health Research, University of Southern Denmark, Campusvej 55, Odense, 5230, Denmark; c Center for Spine Surgery and Research, Lillebaelt Hospital - University Hospital of Southern Denmark, Sygehusvej 24, Kolding, 6000, Denmark

**Keywords:** EQ-5D index value, Health-related quality of life, Response-level mapping, Machine learning, Lumbar spine surgery

## Abstract

**Introduction:**

The transition from EQ-5D-3L to EQ-5D-5L poses challenges for comparing health-related quality of life across studies and clinical settings. Although official EuroQol crosswalk algorithms are commonly used to map between versions, alternative mapping approaches may be relevant for disease-specific populations.

**Research question:**

This study evaluated the performance of machine-learning models for bidirectional mapping between EQ-5D-3L and EQ-5D-5L in patients undergoing evaluation for elective lumbar spine surgery for degenerative conditions.

**Material and methods:**

Paired EQ-5D-3L and EQ-5D-5L single-center responses (n = 766) were analyzed using repeated cross-validation. Response-level mapping was performed using parametric ordinal regression models (VGAM, ordinalNet) and tree-based machine-learning models (XGBoost, CatBoost, ordinalForest). Both hard and soft prediction approaches were evaluated on held-out data and compared with the official crosswalk algorithms using RMSE, MAE, bias (mean signed error), R^2^, and CCC.

**Results:**

Soft predictions generally showed lower prediction errors and higher predictive R^2^ than official crosswalk algorithms, particularly for 3L-to-5L mapping, while improvements were smaller for 5L-to-3L mapping, and CCC favored the official crosswalk algorithms. Hard predictions showed similar improvements for 3L-to-5L mapping, except for CatBoost, but higher errors and lower predictive R^2^ for 5L-to-3L mapping. Differences between machine-learning models were generally small across all evaluation metrics.

**Discussion and conclusion:**

Machine-learning models provided alternative approaches to the official EuroQol crosswalk algorithms, with performance varying by mapping direction and prediction approach. Soft prediction generally showed more favorable predictive performance, particularly for 3L-to-5L mapping. Overall, these findings support further investigation of response-level mapping machine-learning approaches to harmonize mixed-format EQ-5D data.

## Highlights

• Machine-learning models using soft predictions achieved lower prediction errors and higher predictive R^2^ than the official EuroQol crosswalk algorithms for bidirectional EQ-5D-3L/5L mapping in an elective lumbar spine surgery cohort.
• Soft prediction improved prediction errors and accuracy in both mapping directions, whereas hard prediction showed more variable performance depending on the mapping direction and machine-learning model.
• Differences in performance estimates between machine-learning models were generally small within each prediction method and mapping direction.

## 1 Introduction

The EuroQol five-dimensional instrument (EQ-5D) is widely used to measure health-related quality of life (HRQoL) across clinical settings, including spine surgery (Group, 1990). The introduction of the five-level version (EQ-5D-5L) aimed to improve sensitivity and reduce ceiling effects compared with the original three-level version (EQ-5D-3L) (Herdman et al., 2011). However, the coexistence of the two instruments has created challenges for longitudinal analyses and comparisons across datasets that contain both EQ-5D versions. To address this issue, the EuroQol Group developed standardized crosswalk algorithms to map responses between EQ-5D-3L and EQ-5D-5L (Van Hout, 2012). Although these algorithms have been validated in general populations, their performance has been reported to be limited to several disease-specific cohorts, particularly for individual-level predictions (Janssen et al., 2018; Thompson et al., 2020; Ameri et al., 2020; Christiansen et al., 2021). Both versions of the EQ-5D have been evaluated in patients with low back pain (Garratt et al., 2021), but evidence on mapping performance in surgical spine populations remains limited. In a recent study using the same cohort as the present study, Pedersen et al. (2026) evaluated the EuroQol crosswalk algorithms for bidirectional mapping between EQ-5D-3L and EQ-5D-5L (Pedersen et al., 2026). The study found systematic prediction errors, distributional distortion, and limitations in capturing individual-level health-state estimates. In particular, mapping from EQ-5D-3L to EQ-5D-5L resulted in reduced precision. These findings indicate that, although the crosswalk algorithms provide a standardized approach for harmonizing EQ-5D data, their performance may be limited when individual-level estimates are required.

Other studies have also examined differences between value sets derived using crosswalk procedures and those obtained from direct evaluation studies, reporting discrepancies in EQ-5D index values across countries and health states (Khan et al., 2016; Bailey et al., 2025; Hernandez-Alava et al., 2022; Jiang et al., 2022; Golicki et al., 2014). In addition, differences in the distributional properties of the two instruments have been documented, with EQ-5D-5L showing a more unimodal distribution than the typically multimodal distribution observed for EQ-5D-3L (Mulhern et al., 2018).

Since the introduction of the EuroQol crosswalk algorithms, several methodological approaches have been proposed to map between EQ-5D-3L and EQ-5D-5L. These include response-level mapping, which predicts responses to the EQ-5D descriptive system, and utility-level mapping, which directly predicts EQ-5D index values. Previous studies have developed econometric and statistical mapping frameworks to model the relationship between the two instruments (Hernández-Alava et al., 2017; Hernandez Alava et al., 2023; Van Hout and Shaw, 2021; Torkilseng et al., 2025). For example, the study by Hernandez Alava et al. (2023) developed a joint multi-equation ordinal regression model of the ten response items across EQ-5D-3L and EQ-5D-5L, enabling bidirectional mapping within a unified statistical framework that is consistent with the observed joint distribution of responses (Hernandez Alava et al., 2023).

Despite these methodological developments, it remains unclear whether flexible predictive modeling approaches can improve individual-level mapping between EQ-5D-3L and EQ-5D-5L compared with the established crosswalk algorithms. Machine-learning models may be particularly suited for response-level prediction, as they can capture complex, non-linear relationships and interactions between EQ-5D dimensions while preserving the structure of the descriptive system and allowing index values to be calculated using any available country-specific value set. To our knowledge, no prior study has compared response-level machine learning models with the official EuroQol crosswalk algorithms in a lumbar spine surgery cohort.

The present study builds on Pedersen et al. (2026) by using the same 766 paired EQ-5D-3L/EQ-5D-5L observations to evaluate response-level machine-learning models for bidirectional mapping and compare their performance with the EuroQol crosswalk algorithms. We also examine whether soft versus hard predictions affect mapping performance.

We hypothesized that response-level machine-learning models could improve predictive accuracy, error distribution, and agreement with observed EQ-5D index values compared with the EuroQol crosswalk algorithms. If successful, such models could provide a flexible alternative for harmonizing EQ-5D responses in clinical registries and research datasets where mixed versions are unavoidable.

## 2 Methods

### 2.1 Study design

In this retrospective cohort study, patient-reported outcome measure (PROM) data from 766 adult patients undergoing evaluation for elective lumbar spine surgery for degenerative conditions were analyzed. Data were originally collected prospectively as part of a single-center observational cohort study conducted by the same authors (Pedersen et al., 2026). The EQ-5D-5L was administered prior to surgical assessment, and the EQ-5D-3L at the time of surgical scheduling. All participants were 18 years or older and had completed both instruments, with up to 90 days between administrations. Participants with incomplete EQ-5D responses were excluded. The clinical characteristics of the study population have been described elsewhere (Pedersen et al., 2026).

### 2.2 Data preprocessing

The EQ-5D items were prepared for use in machine-learning models by encoding responses as ordered categorical variables and structuring them according to the corresponding EQ-5D dimensions. The EQ-5D index values from the EQ-5D-3L were calculated using the value set derived by Dolan et al. (2002), and index values from the EQ-5D-5L were calculated using the value set derived by Devlin et al. (2018). The crosswalk and reverse crosswalk EQ-5D index values were calculated using the EuroQol crosswalk algorithms (van et al., 2012). All EQ-5D index values were derived using the UK value set for EQ-5D-3L and the English value set for EQ-5D-5L. Unique EQ-5D health states were enumerated for both EQ-5D-3L and EQ-5D-5L systems, and empirical frequency distributions were derived to characterize response heterogeneity in the study sample. Inter-dimensional relationships between EQ-5D domains were assessed using Pearson correlation coefficients based on numeric re-coding of ordinal responses.

### 2.3 Machine-learning models

A set of machine-learning models was compared with the EuroQol crosswalk algorithms for mapping between the EQ-5D-3L and EQ-5D-5L instruments. The evaluated models comprised both parametric ordinal regression–based approaches, including vector generalized linear and additive models (VGAM) and penalized ordinal regression (ordinalNet), and non-parametric, tree-based approaches, including eXtreme Gradient Boosting (XGBoost), Categorical Boosting (CatBoost), and random forest–based ordinal regression (ordinalForest). All models were fitted and implemented using publicly available software.

In preliminary analyses, additional machine-learning approaches, including regression-, kernel-, and neural network–based models, were explored to assess their suitability for the mapping task. However, many of these models are primarily designed for continuous outcomes and do not explicitly account for the ordered categorical structure of EQ-5D responses.

The models selected for the final analysis represent complementary methodological approaches for predicting ordered categorical outcomes and are commonly used in health outcomes research and applied machine learning. The models support ordinal or multi-class outcomes and can therefore be applied directly to EQ-5D response-level data.

VGAM and ordinalNet use cumulative link ordinal regression that explicitly accounts for the ordered nature of EQ-5D response levels. These models assume an underlying continuous latent variable and estimate category probabilities within a parametric ordinal framework.

XGBoost and CatBoost were implemented as multiclass classification algorithms that model complex nonlinear relationships and interactions among EQ-5D dimensions without explicitly imposing ordinality in the loss function. For XGBoost, monotonic constraints were additionally specified to preserve the expected ordinal relationship between worsening EQ-5D response levels and predicted outcomes. The ordinalForest extends the random forest framework to ordinal outcomes by incorporating ordering information into the tree construction process.

Across all models, EQ-5D response levels were encoded as ordered factors and mapped to numeric representations for model training. For probabilistic models, predicted class probabilities were subsequently transformed into EQ-5D index values using expected value aggregation over the EQ-5D value sets.

### 2.4 Model evaluation and statistical analysis

Mapping was performed at the level of the EQ-5D descriptive system by predicting responses for each dimension, with index values subsequently calculated using the corresponding value sets. The EuroQol crosswalk algorithms are also based on probabilistic relationships between EQ-5D response profiles, but they directly generate expected EQ-5D index values rather than predicted response levels. All models were applied to the same response-level input data and evaluated using consistent prediction and evaluation procedures.

To assess the impact of accounting for predictive uncertainty on model performance, both hard and soft prediction approaches were evaluated. Hard prediction assigns the response level with the highest predicted probability for each EQ-5D dimension and calculates the corresponding EQ-5D index value. In contrast, soft prediction uses the predicted probabilities for all possible response levels to calculate a probability-weighted average EQ-5D index value. Models capable of producing class probability distributions (VGAM, ordinalNet, XGBoost, and CatBoost) were evaluated using both approaches. The model ordinalForest was evaluated using only hard ordinal predictions, as it does not provide calibrated class probability estimates.

Model performance was evaluated using repeated k-fold cross-validation with out-of-fold predictions. The dataset was randomly partitioned into five folds, and the procedure was repeated ten times with different random splits. To ensure stable model estimation for infrequent response levels, rare health states were retained in the training sets across all folds. One response level in the EQ-5D-5L pain/discomfort dimension occurred only once in the dataset. To avoid fold-specific model-fitting failures due to missing response levels, this observation was retained in the training data across cross-validation iterations. Given the negligible prevalence of this response level, any resulting optimism in overall performance estimates is expected to be minimal.

The mapping performance of all models was compared with the EuroQol crosswalk algorithms using root mean squared error (RMSE), mean absolute error (MAE), bias (mean signed error), predictive R^2^, and concordance correlation coefficient (CCC). These performance metrics were selected to provide a common evaluation framework for comparing the machine-learning models and crosswalk algorithms.

To quantify uncertainty in the differences between machine-learning models and the crosswalk algorithms, paired bootstrap resampling was performed at the patient level. For each bootstrap sample, all performance metrics were calculated using the same resampled observations, and the difference was calculated as the model performance minus the crosswalk performance. Ninety-five percent confidence intervals for the paired differences were obtained from the 2.5th and 97.5th percentiles of 5000 bootstrap samples.

In addition, the distributions of predicted EQ-5D index values were visually assessed with density plots, and agreement with the best-performing model was evaluated with Bland–Altman plots. All analyses were performed using R version 4.5.2 (Team, 2020) and the R packages eq (5d) (Morton et al., 2026), VGAM (Yee, 2015), xgboost (Chen et al., 2015), ordinalForest (Hornung, 2020), ordinalNet (Wurm et al., 2021), and catboost (Dorogush et al., 2018). The models were fitted using the default hyperparameter settings specified by the R packages.

## 3 Results

A total of 56 (23%) health states were observed for EQ-5D-3L out of 243 possible health states, while 285 (9.1%) health states were observed for EQ-5D-5L out of 3125 possible health states. The most frequent health states for EQ-5D-3L were 21221 (17.23%), followed by 22221 (8.22%), 22332 (6.53%), and 11221 (6.40%). For EQ-5D-5L, the most common health states were 42442 (2.35%), followed by 42441 (1.70%), 43443 (1.70%), and 43442 (1.70%). The lower proportion of observed EQ-5D-5L health states reflects the substantially larger number of possible health-state combinations in the five-level descriptive system.

Inter-dimensional correlations for the EQ-5D-3L items were generally low to moderate, ranging from 0.10 to 0.34, with stronger correlations observed between physical domains (mobility, self-care, activity, and pain) and weaker correlations involving anxiety and depression. A similar pattern was observed for EQ-5D-5L, although correlations were generally higher, ranging from 0.26 to 0.53 (see Appendix Figure A1).

Model performance is presented in Table 1, Table 2 for response-level mapping between EQ-5D-5L and EQ-5D-3L, using either hard or soft prediction where applicable. No single model consistently performed best across all metrics and mapping directions. The models ordinalNet and VGAM generally showed the lowest prediction errors, although differences between models were small and varied across metrics. For mapping from EQ-5D-5L to EQ-5D-3L, ordinalNet achieved an RMSE of 0.27, MAE of 0.23, bias of −0.01, R^2^ of 0.30, and CCC of 0.46, while VGAM achieved an RMSE of 0.27, MAE of 0.22, bias of −0.01, R^2^ of 0.29, and CCC of 0.48. For mapping from EQ-5D-3L to EQ-5D-5L, ordinalNet achieved an RMSE of 0.21, MAE of 0.17, bias of 0.002, R^2^ of 0.34, and CCC of 0.50, while VGAM achieved an RMSE of 0.21, MAE of 0.17, bias of 0.001, R^2^ of 0.34, and CCC of 0.51.

**Table 1:**
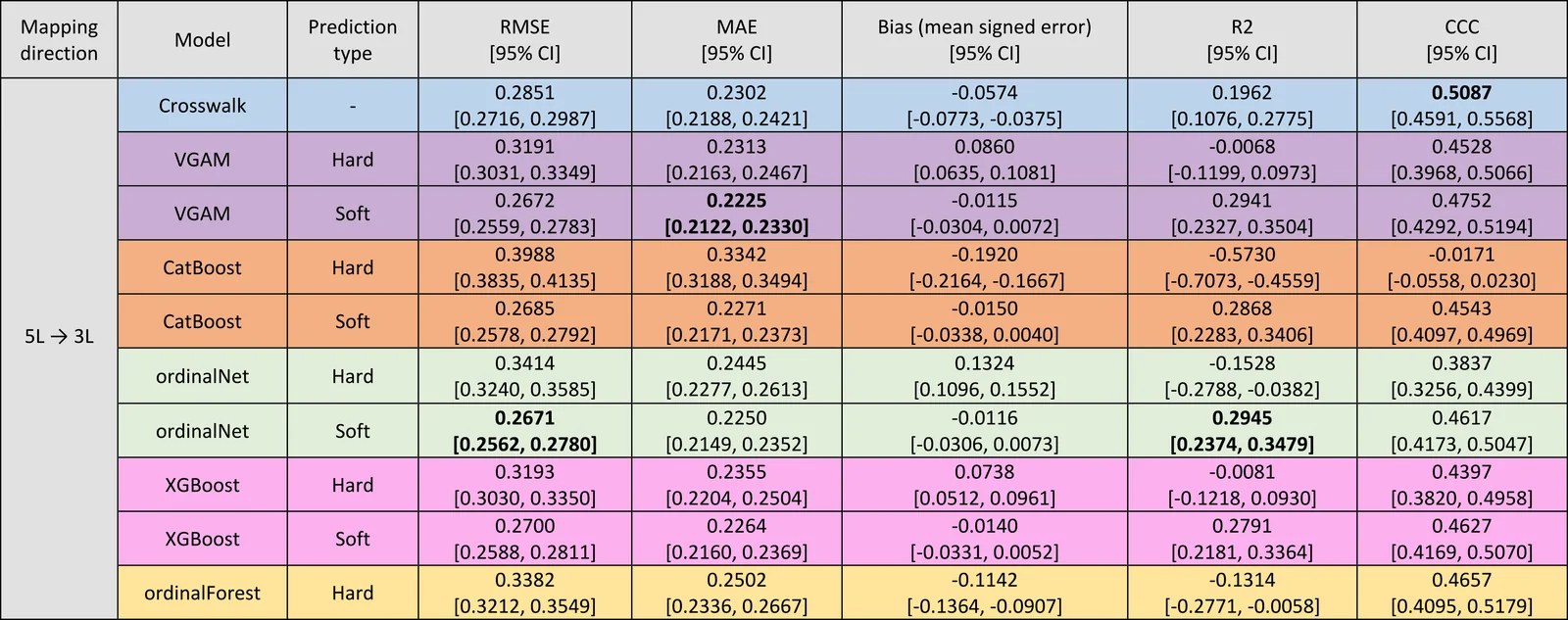
Performance metrics for the EuroQol crosswalk algorithm and machine-learning models for mapping from EQ-5D-5L to EQ-5D-3L.

RMSE = root mean squared error; MAE = mean absolute error; R^2^ = predictive R-squared.

CCC = concordance correlation coefficient. Best-performing values (lowest RMSE/MAE and highest R^2^/CCC) are highlighted.

Values are presented with 95% confidence intervals.

**Table 2:**
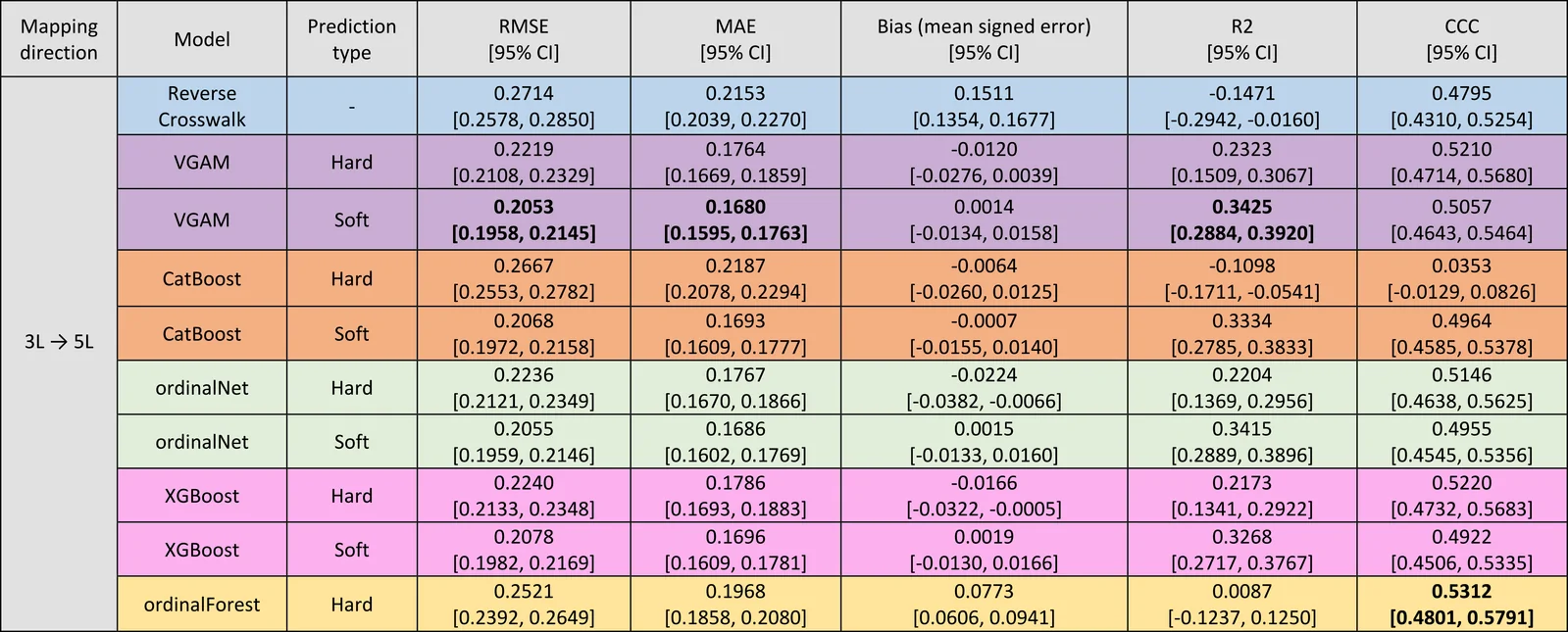
Performance metrics for the EuroQol crosswalk algorithm and machine-learning models for mapping from EQ-5D-3L to EQ-5D-5L.

RMSE = root mean squared error; MAE = mean absolute error; R^2^ =predictive R-squared.

CCC = concordance correlation coefficient. Best-performing values (lowest RMSE/MAE and highest R^2^/CCC) are highlighted.

Values are presented with 95% confidence intervals.

Bias ranged from −0.19 to 0.13 for the mapping from EQ-5D-5L to EQ-5D-3L and from −0.02 to 0.15 for the mapping from EQ-5D-3L to EQ-5D-5L, indicating generally small systematic differences between predicted and observed EQ-5D index values. For measures of explained variance and agreement, performance varied across models and mapping directions. The models, ordinalNet and VGAM achieved the highest R^2^ values for the EQ-5D-5L to EQ-5D-3L (0.30) and EQ-5D-3L to EQ-5D-5L (0.34) mappings, respectively.

The performance of the machine-learning models relative to the crosswalk algorithms is shown in Table 3. With soft prediction, all machine-learning models showed lower prediction errors (RMSE and MAE) and higher predictive R^2^ than the official EuroQol crosswalk algorithms in both mapping directions. For CCC, however, the crosswalk showed stronger agreement for mapping from EQ-5D-5L to EQ-5D-3L. With hard prediction, performance varied across metrics and mapping direction.

**Table 3:**
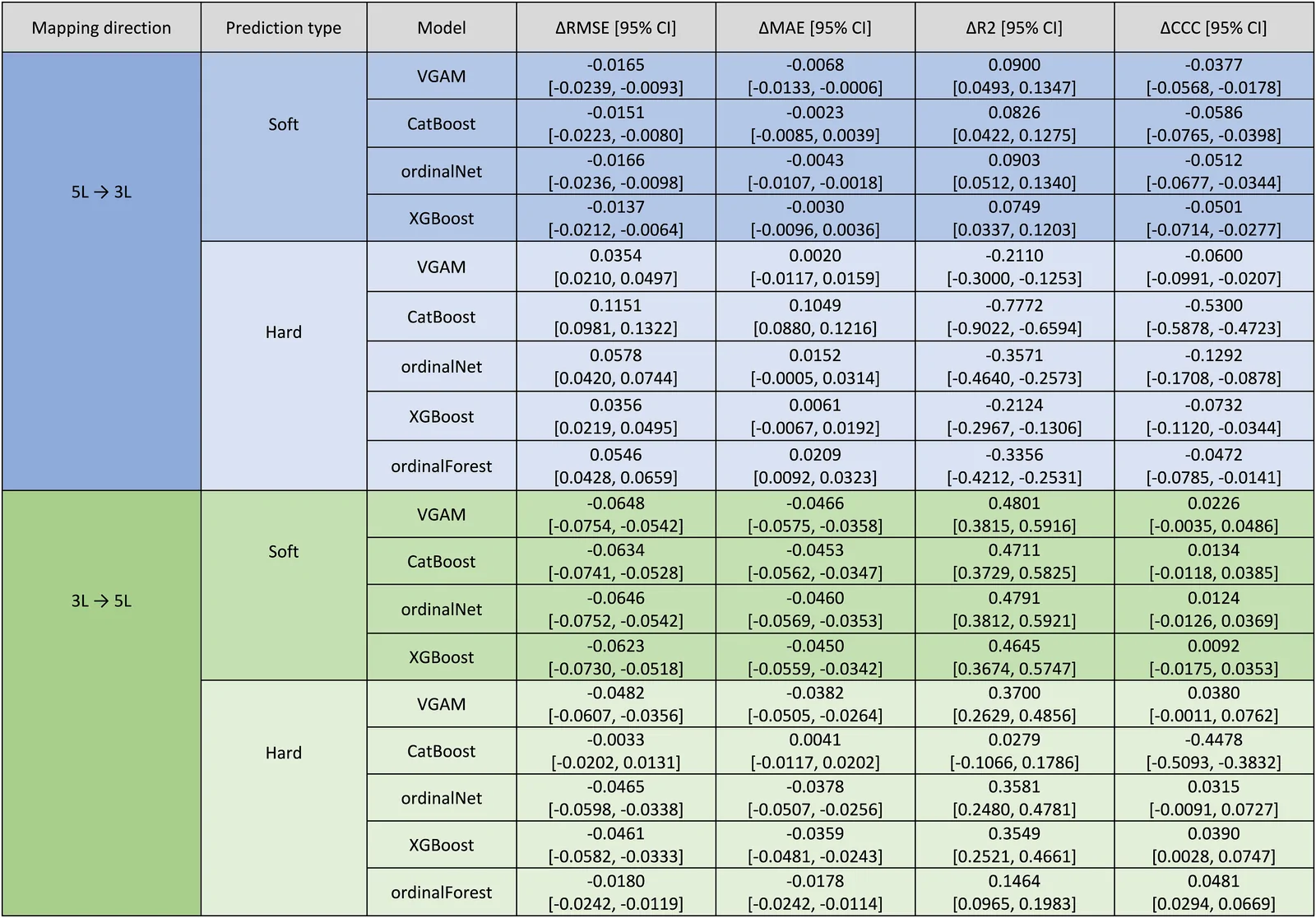
Paired differences in performance between machine-learning models and the EuroQol crosswalk algorithms.

Δ = machine-learning model − EuroQol crosswalk algorithm. Negative values indicate lower RMSE and MAE for the machine-learning model, whereas positive values indicate higher R^2^ and CCC. Values are presented as differences with 95% confidence intervals.

The distributions of predicted EQ-5D index values varied across models and prediction approaches. Visual inspection suggested that, for mapping from EQ-5D-5L responses to EQ-5D-3L index values, hard prediction more closely resembled the observed distribution of EQ-5D index values than soft prediction (Fig. 1, Fig. 2). In contrast, for mapping from EQ-5D-3L responses to EQ-5D-5L index values, soft prediction showed better agreement with the observed distribution than hard prediction (Fig. 3, Fig. 4). Furthermore, under soft prediction (Fig. 1, Fig. 3), all machine-learning models produced similar distributions of predicted EQ-5D index values, whereas greater variation in distributional shape was observed across models when using hard prediction (Fig. 2, Fig. 4).

**Fig. 1:**
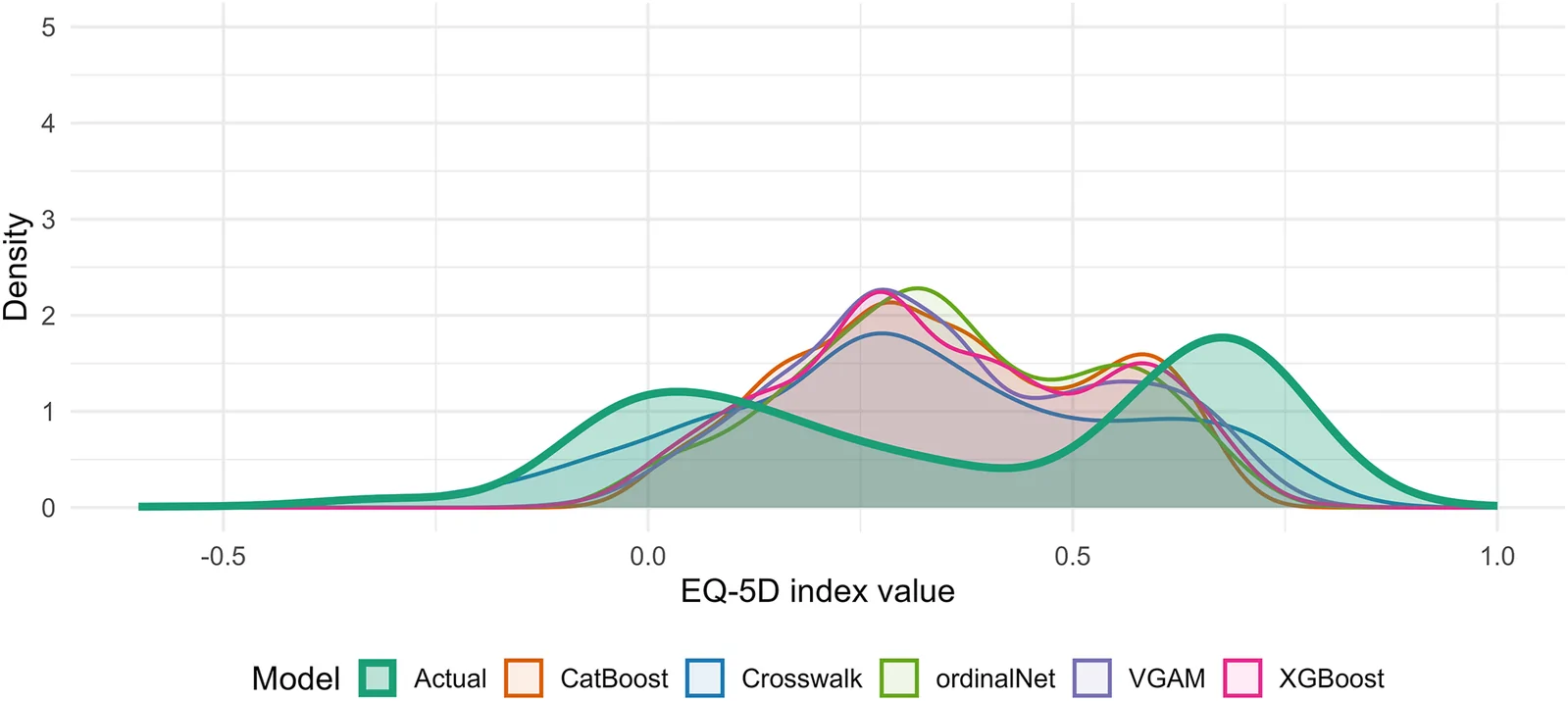
Distribution of EQ-5D-3L Index Values using Soft Prediction on 5L to 3L Response Items; Actual = observed index values.

**Fig. 2:**
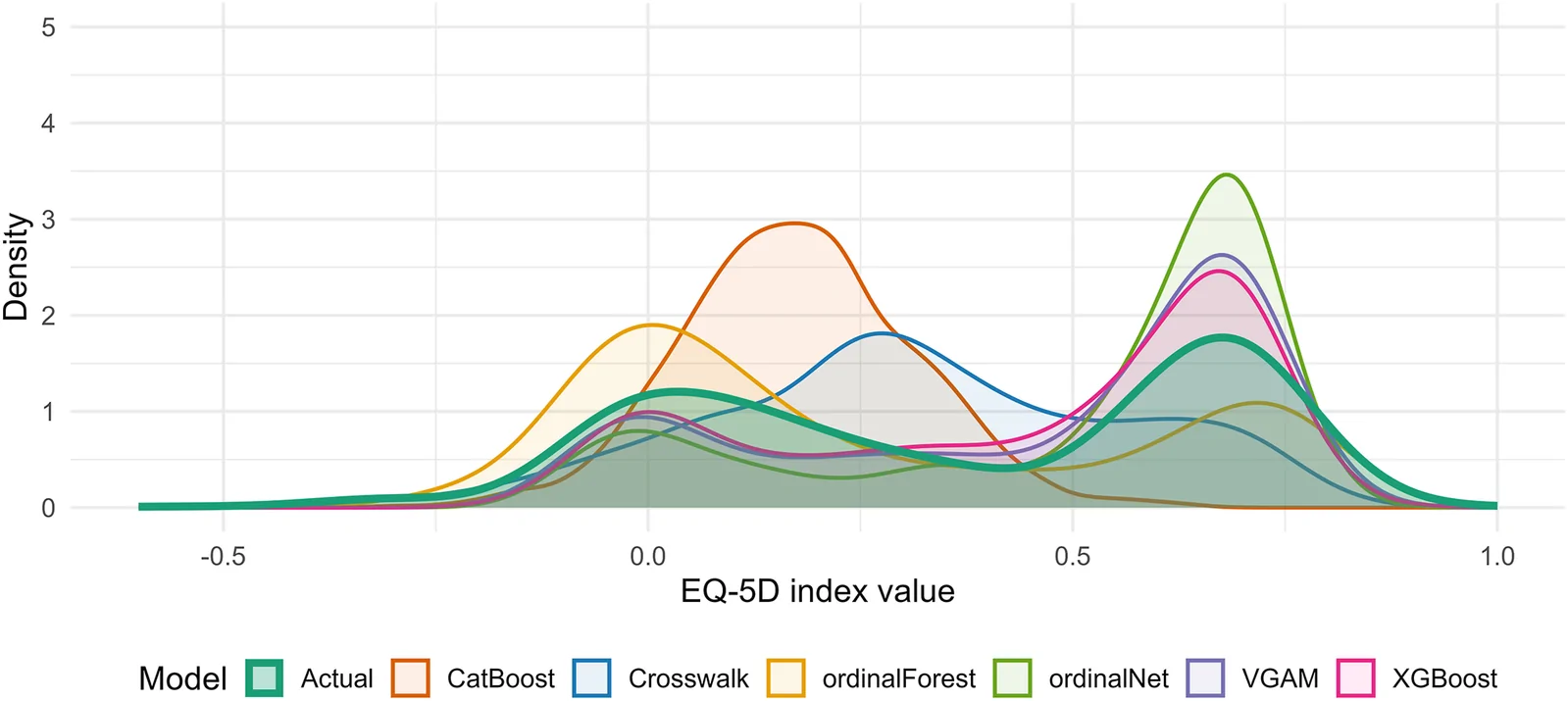
Distribution of EQ-5D-3L Index Values using Hard Prediction on 5L to 3L Response Items; Actual = observed index values.

**Fig. 3:**
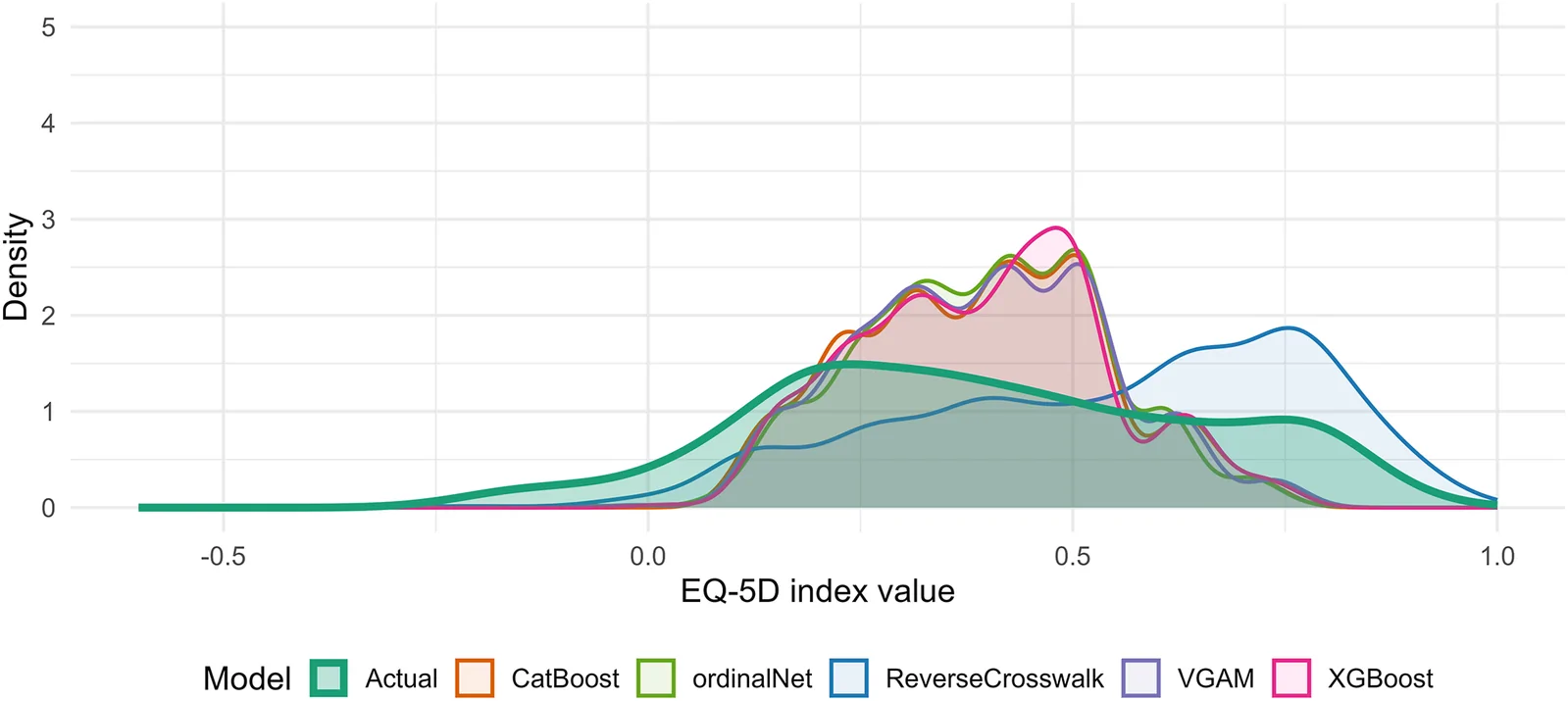
Distribution of EQ-5D-5L Index Values using Soft Prediction on 3L to 5L Response Items; Actual = observed index values.

**Fig. 4:**
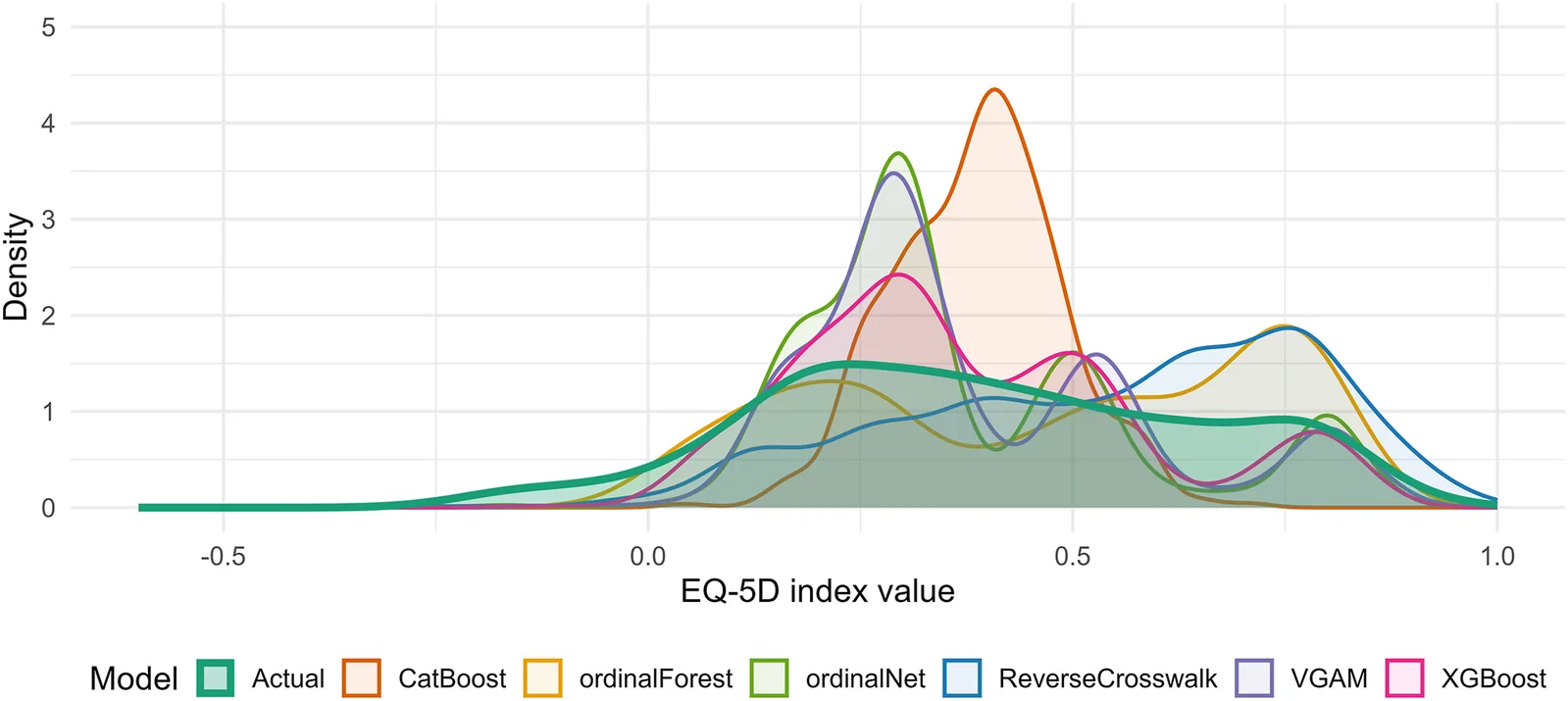
Distribution of EQ-5D-5L Index Values using Hard Prediction on 3L to 5L Response Items; Actual = observed index values.

Compared with the official EuroQol crosswalk algorithms, the machine-learning models showed no consistent advantage in reproducing the observed distribution of EQ-5D index values. Agreement with the observed distribution depended on both the mapping direction and whether hard or soft prediction was used.

The Bland–Altman plots for VGAM using soft predictions illustrate small mean differences but relatively wide limits of agreement in both mapping directions, indicating proportional bias with overestimation of lower index values and underestimation of higher index values (Fig. 5, Fig. 6). The clustering of observations reflects the discrete nature of EQ-5D index values. There was some evidence of heteroscedasticity, with slightly greater dispersion at higher mean index values.

**Fig. 5:**
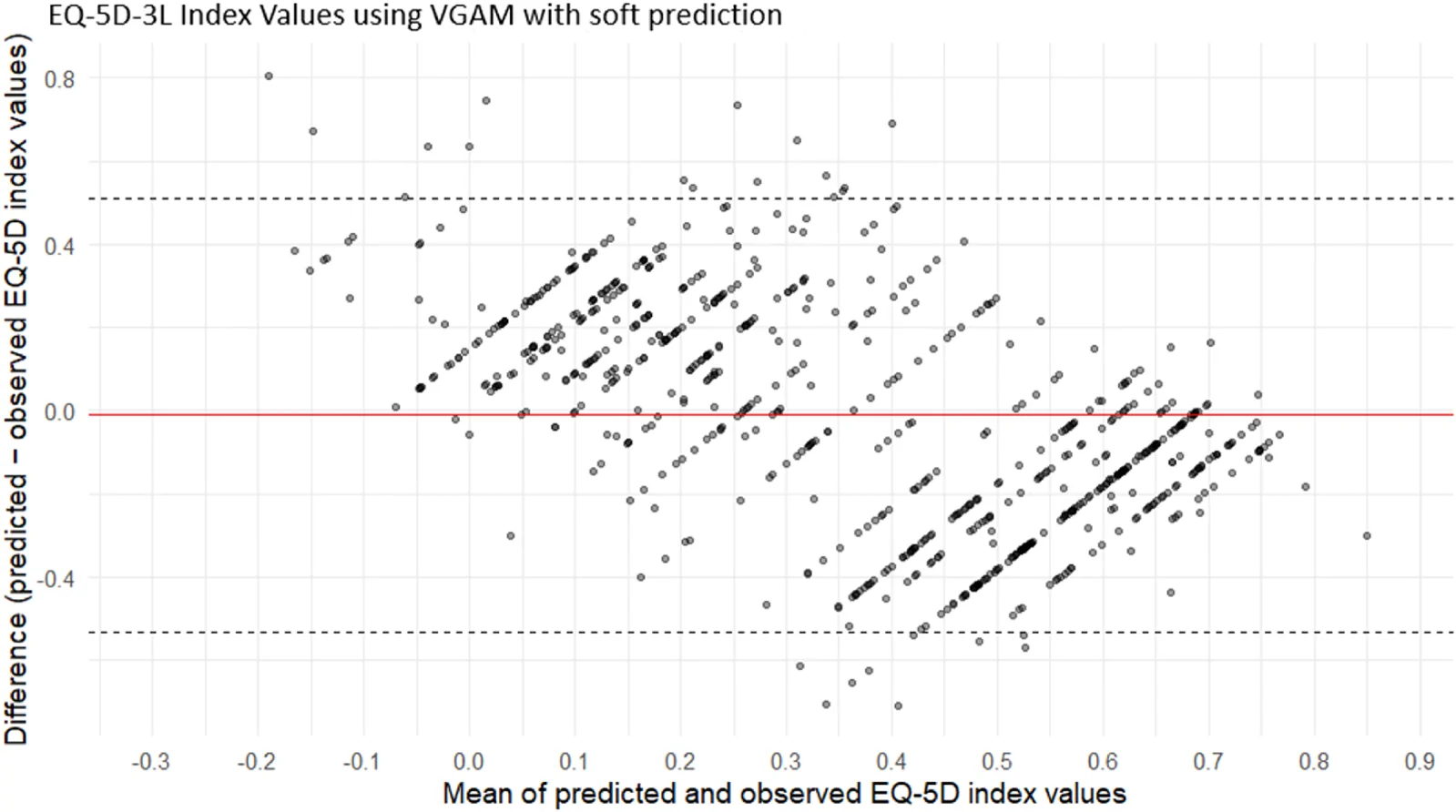
Bland-Altman plot of the predicted EQ-5D-3L index values using VGAM with soft prediction vs the observed EQ-5D-3L index values.

**Fig. 6:**
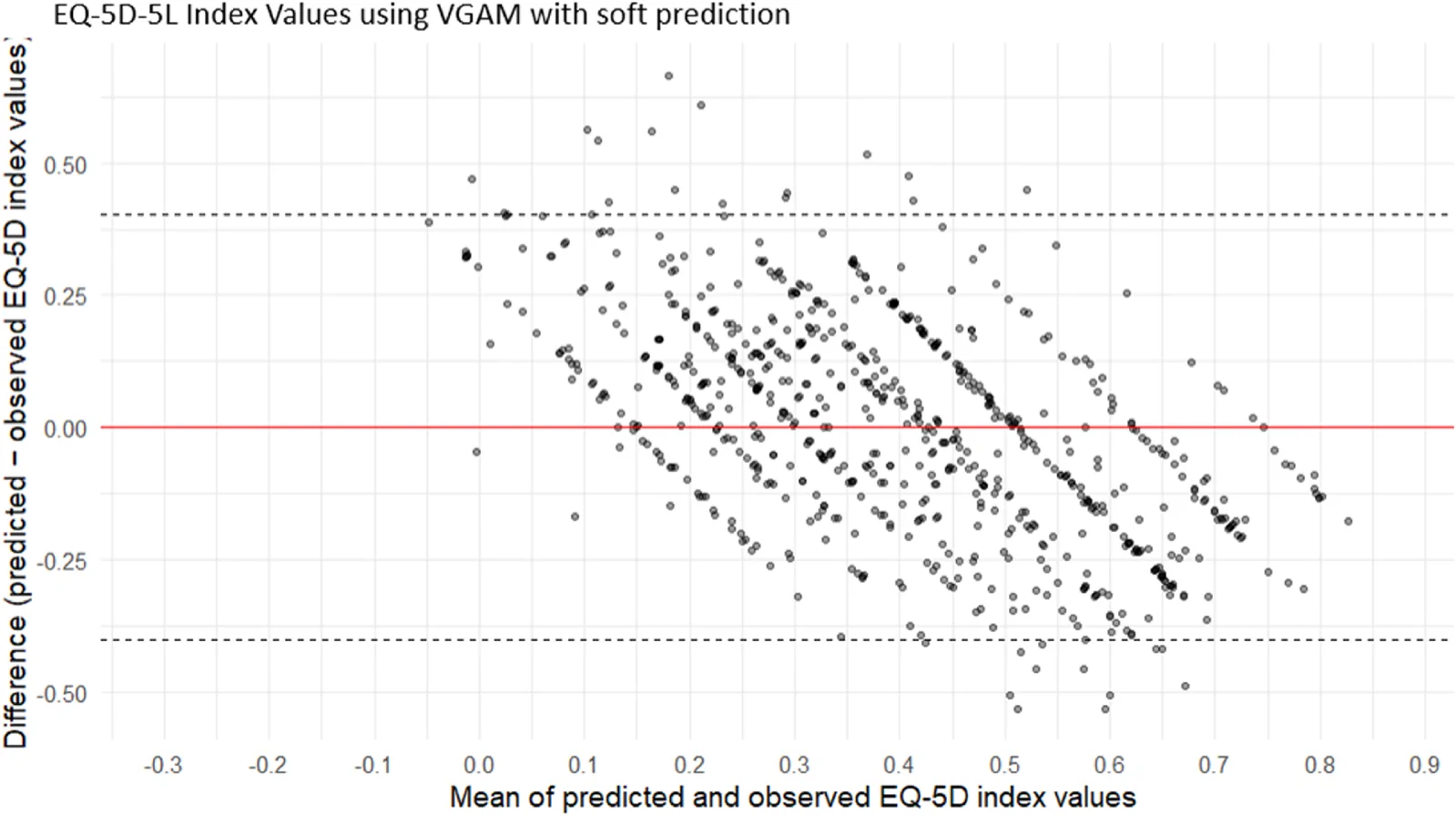
Bland-Altman plot of the predicted EQ-5D-5L index values using VGAM with soft prediction vs the observed EQ-5D-5L index values.

Although mean differences were small, the limits of agreement were relatively wide.

## 4 Discussion

In this study, machine-learning models with soft predictions showed lower prediction errors and higher predictive R^2^ compared to the EuroQol crosswalk algorithm in mapping from EQ-5D-5L to EQ-5D-3L in patients scheduled for elective lumbar spine surgery. This suggests that incorporating probabilistic information about response categories better captures the uncertainty inherent in mapping between descriptive systems with different levels of granularity. In contrast, hard predictions showed more variable performance depending on the mapping direction and machine-learning model when compared to the crosswalk algorithm, likely due to loss of distributional information when collapsing predictions into a single response item.

For the reverse mapping from EQ-5D-3L to EQ-5D-5L, most models also achieved lower prediction errors than the reverse crosswalk algorithm, although performance improvements were less consistent for CatBoost and smaller for ordinalForest using hard prediction, indicating that some models may struggle more when expanding from a less detailed to a more detailed descriptive system. However, differences in performance across models were generally small, with most approaches demonstrating broadly comparable predictive performance across evaluation metrics.

In addition to the EuroQol crosswalk algorithms, alternative approaches have been proposed to map between EQ-5D-3L and EQ-5D-5L, including utility-level mapping methods implemented in functions such as *eq (5)dmap* in *eq (5d)* R package. These approaches directly estimate EQ-5D index values rather than predicting responses to the descriptive system. While such methods may provide accurate predictions for specific value sets, response-level mapping preserves the underlying health-state structure and allows predicted responses to be valued using different country-specific tariffs. This is supported by previous work by Hernández-Alava et al. (2017) (Hernández-Alava et al., 2017). For this reason, the present study focused on response-level prediction.

Despite overall improvements in predictive performance, visual inspection of the predicted EQ-5D index value distributions revealed differences between soft and hard prediction that were not fully reflected in the error-based metrics. Hard prediction sometimes produced distributions that more closely resembled the observed EQ-5D index values, whereas soft prediction yielded smoother distributions with reduced variability. However, soft prediction consistently achieved lower RMSE and MAE values. This apparent discrepancy reflects the distinction between distributional fit and pointwise prediction accuracy. Hard prediction prioritizes the most likely health state and may therefore better reproduce the empirical distributional shape, whereas soft prediction incorporates the full predicted probability distribution and yields an estimate of the expected EQ-5D index value. When the target of interest is the expected health utility, soft prediction is therefore theoretically preferable, as it propagates uncertainty across the predicted health-state distribution rather than conditioning on a single most likely outcome. This may explain why soft prediction performed better in RMSE- and MAE-based evaluations, even when it appeared less aligned with the empirical distribution of observed index values.

The selected models differ in their ability to capture nonlinear relationships, interactions, and the ordinal structure of EQ-5D responses. Parametric approaches, such as VGAM and ordinalNet, explicitly account for the ordered nature of response items through cumulative link ordinal regression models based on an underlying latent variable, but they rely on linear-predictor assumptions, which may limit their flexibility. In contrast, tree-based models, including XGBoost, CatBoost, and ordinalForest, can capture complex nonlinear relationships and interactions between EQ-5D dimensions but do not explicitly impose the ordinal structure within the model formulation. Instead, these relationships are learned implicitly through the predictor set.

These findings should be interpreted in light of the systematic deviations observed in the present study, in which several models tended to overestimate lower index values and underestimate higher ones. This pattern was also evident in the Bland–Altman analyses, which demonstrated proportional bias across the range of EQ-5D index values.

Similar patterns have been reported in previous mapping studies. For example, Khan et al. (2016) (Khan et al., 2016) found larger prediction errors in poorer health states when mapping between EQ-5D instruments. Likewise, Bailey et al. (2025) (Bailey et al., 2025) reported systematic differences and limited agreement between crosswalk- and valuation-based EQ-5D value sets, with Bland–Altman analyses indicating structured discrepancies across health states.

These challenges may partly reflect inherent differences in the descriptive systems, particularly the coarser structure of EQ-5D-3L, which provides fewer response levels and may limit sensitivity at the extremes of health. Taken together, this suggests that accurately capturing extreme health states remains difficult across modeling approaches and that proportional bias persists in EQ-5D mapping.

In this study, the UK EQ-5D-3L and England EQ-5D-5L value sets were applied. Although the data were collected in a Danish population, these tariffs were used to facilitate comparability with existing crosswalk studies and methodological research, which commonly rely on UK-based valuations. As the modeling framework predicts responses at the descriptive-system level, alternative country-specific value sets can be applied without re-estimating the models. However, predictive performance and resulting distributions of EQ-5D index values may vary depending on population characteristics and valuation context.

Previous research has highlighted that differences between national value sets can influence estimated EQ-5D index values. For example, Torkilseng et al. (2025) (Torkilseng et al., 2025) demonstrated that the Danish EQ-5D-5L index values can be approximated from UK EQ-5D-3L utilities at an aggregate level, although such approaches may not capture individual-level variation. In addition, clinically meaningful differences in EQ-5D-5L index values have been established in a lumbar spine surgery population, as shown by Yakdan et al. (2025) (Yakdan et al., 2025), underscoring the importance of accurate prediction at the individual level.

### 4.1 Limitations

Several limitations should be considered when interpreting the findings. The EQ-5D responses were collected in different clinical settings, with EQ-5D-5L administered in the medical spine department and EQ-5D-3L in the surgical spine department. As patients were typically referred from the medical to the surgical department, changes in clinical status or treatment effects may have occurred between administrations. Although the maximum interval between assessments was 90 days, the exact time between completion of the two instruments varied across patients. In patients with degenerative spinal conditions, health status may change during this period, meaning that differences in observed EQ-5D responses may reflect both instrument-related differences and true changes in health-related quality of life. This temporal discrepancy may therefore introduce additional error into the mapping models and make it difficult to attribute all observed prediction errors to the mapping procedure itself. The fixed order of administration (EQ-5D-5L followed by EQ-5D-3L) may also have introduced recall, anchoring, or response consistency effects, which have previously been shown to influence EQ-5D responses in within-person comparisons (Hernandez-Alava et al., 2022).

While repeated k-fold cross-validation was used to improve robustness, the observed health-state space was sparse relative to the number of possible combinations. Only 56 (23%) of the 243 possible EQ-5D-3L health states and 285 (9.1%) of the 3125 possible EQ-5D-5L health states were observed. Consequently, the models, particularly the tree-based models, had limited empirical information for some rare or unobserved health states, which may reduce the reliability of predictions for such health states and limit generalizability to populations with substantially different health-state distributions. In addition, one response level in the pain/discomfort dimension occurred only once. Although this observation was retained in the training sets to avoid model instability during cross-validation, the presence of rare health states may still limit the generalizability of the models and introduce some degree of optimism in performance estimates. Finally, response-level models were estimated within a single-center cohort, and external validation in independent datasets is required to assess generalizability across different populations and value sets.

### 4.2 Future directions

Although EQ-5D-5L has largely replaced EQ-5D-3L in many clinical and health economics applications, EQ-5D-3L continues to be used in certain contexts, particularly in longitudinal studies and ongoing trials where switching instruments during follow-up may compromise comparability.

Future studies should investigate whether EQ-5D-5L responses can be predicted from EQ-5D-3L responses in longitudinal datasets, accounting for the time interval between administrations, which may influence reported health states and societal preferences across EQ-5D dimensions. Additionally, future studies should investigate whether incorporating patient characteristics, such as demographic information or other patient-reported outcomes, improves response-level mapping. Previous studies have shown that including age and sex reduced information criteria but did not improve predictive accuracy in utility-level models (Van Hout and Shaw, 2021). It remains unclear whether these covariates could enhance response-level mapping.

## 5 Conclusion

In this lumbar spine surgery cohort, several machine-learning models achieved lower prediction errors than the official EuroQol crosswalk algorithms for bidirectional mapping between EQ-5D-3L and EQ-5D-5L. Performance gains were most consistent with soft prediction, particularly for mapping from EQ-5D-3L to EQ-5D-5L, whereas improvements for EQ-5D-5L to EQ-5D-3L were smaller and depended on the performance metric evaluated.

Overall, response-level machine-learning approaches offer a promising alternative to the official EuroQol crosswalk algorithms, improving predictive accuracy while preserving the ability to model EQ-5D responses at the descriptive-system level. However, external validation is required, and further work should address limitations related to timing effects, distributional bias, and modeling of joint response dependence before broader application.

## Author contributions

Natalie Hong Siu Chang: Conceptualization, Visualization, Methodology, Formal analysis, Data curation, Writing – original draft. Casper Friis Pedersen: Conceptualization, Data curation, Methodology, Writing – review and editing. Mikkel Østerheden Andersen: Conceptualization, Writing – review and editing. Leah Yacat Carreon: Conceptualization, Supervision, Validation, Writing – review and editing. All authors have approved the final article.

## Data availability

Application forms to use the described data for research projects are available from the Spine Centre of Southern Denmark [contact: forskning.rygcenter@rsyd.dk]. The code used for the analysis is available from the corresponding author upon reasonable request.

## Declaration of competing interest

The authors declare that they have no known competing financial interests or personal relationships that could have appeared to influence the work reported in this paper.
