# Supplementary material for: Mapping between EQ-5D-5L and EQ-5D-3L in lumbar spine surgery patients: Machine learning compared to crosswalk algorithms
Source: Brain Spine. 2026 Sep 28;6:107459. doi: 10.1016/j.bas.2026.107459 (PMC13659494; doi:10.1016/j.bas.2026.107459)
Supplement: Supplementary file 1 [file mmc1.docx]

# Appendix

Figure A1: Inter-dimensional correlations for EQ-5D-3L items (left) and EQ-5D-5L items (right)
